# The Ubiquitin Ligase UBE3A Dampens ERK Pathway Signalling in HPV E6 Transformed HeLa Cells

**DOI:** 10.1371/journal.pone.0119366

**Published:** 2015-03-27

**Authors:** Elisa Aguilar-Martinez, Claire Morrisroe, Andrew D. Sharrocks

**Affiliations:** Faculty of Life Sciences, University of Manchester, Michael Smith Building, Oxford Road, Manchester, M13 9PT, United Kingdom; German Cancer Research Center, GERMANY

## Abstract

Signalling through the ERK MAP kinase pathway plays an important role in many biological processes and it is often deregulated in disease states such as cancer. One major effect of MAP kinase signalling is to promote gene expression through the phosphorylation and activation of transcription factors like ELK1. ELK1 in turn controls the activity of immediate-early genes such as *FOS*. Here we have used ELK1 activation in HeLa cells as a read out to conduct a genome-wide siRNA screen to identify negative regulators of ERK-mediated immediate-early gene activation. One of the candidates that we identified was the E3 ubiquitin ligase UBE3A/E6-AP. Reductions in UBE3A levels cause increased basal levels of ERK activity, a loss of growth factor-mediated ERK activation and concomitant defects in immediate-early gene expression. Thus, UBE3A acts to dampen down basal level ERK activation and to prime the pathway for growth factor-mediated activation. Mechanistically, we demonstrate that UBE3A functions in HeLa cells through its binding partner, HPV18 E6 protein and the E6 target protein p53. Loss of either E6 or p53 blocks the effect of UBE3A depletion on ERK pathway signalling, indicating that in the context of oncogenic viral protein expression, UBE3A plays an important role in negating the consequences of p53 activation on ERK pathway signalling.

## Introduction

The ERK MAP kinase pathway plays an important role in many biological processes including cell growth, neuronal functions, cellular differentiation and development [1]. Furthermore, this pathway is often deregulated in cancer and disease states which affect brain function [2]. Once activated, ERK translocates to the nucleus where it executes one of its major effects in controlling gene transcription [3]. One major class of genes that are activated in response to transient ERK signalling are the immediate-early genes (IEGs), such as *FOS* and *EGR2*. To do this, ERK targets DNA binding transcription factors through phosphorylation and one such target is ELK1 [4]. Although a lot is known about ERK-mediated ELK1 activation, comparatively less is known about the inactivation mechanisms which extinguish ELK1 activity and hence immediate-early gene induction. ELK1 is itself deactivated through dephosphorylation by the phosphatase PP2Bγ/calcineurin [5]. However, there are other inactivation mechanisms employed, including SUMO-mediated recruitment of a histone deacetylase complex to ELK1 which acts locally on chromatin to suppress transcriptional activity [6]. Here we conducted a genome-wide siRNA screen in HeLa cells to identify novel negative regulators of ERK-mediated gene expression by using ELK1 transactivation ability as a molecular readout.

One of the hits that we identified in our screen was the E3 ubiquitin ligase UBE3A. UBE3A was originally identified as a binding partner for the HPV 18 and 16 E6 oncogenic protein [7] and was later shown to act through E6 to promote p53 degradation [8]. UBE3A mutations are associated with neurological defects in humans with Angelman syndrome [9]. Here, we show that loss of UBE3A in HeLa-derived cells caused enhanced ELK1 transactivation and enhanced basal level activity of ELK1-controlled immediate-early genes. This increased activity was attributable to increased basal levels of ERK activation. Growth factor stimulation was unable to further potentiate ERK activation levels and immediate-early gene activation. Mechanistically, UBE3A acted through its binding partner, the oncogenic HPV18 E6 protein, and the E6 target protein p53. Here UBE3A acts to dampen down ERK activity through removing the p53 tumour suppressor protein.

## Materials and Methods

### Plasmid constructs

Gal4-FRT/TO (pAS4014) was created by subcloning the Gal4 coding fragment from pCMV-Gal4 (pAS2071) into the HindIII/BamHI sites of pcDNA5/FRT/TO (Invitrogen). pAS4015, encoding Gal-ELK1 full length was constructed by inserting full length ELK1 from pCMV-Gal-ELK1 (pAS2071) into the BamHI site in Gal4-FRT/TO (pAS4014). G5E1bLucPur (pAS4021) was created by ligating BglII/NheI-cleaved PCR product (primers ADS2577 and ADS2578, template pAS2451) into the same sites in pGL4.20(Luc2/Puro) (Promega). eGFP-ERK2 (pAS4176) and pcDNA3-MEK (pAS4177) have been described previously (kindly provided by Rony Seger [10]).

### Tissue culture, transfections and reporter assay

**Fig 1:**
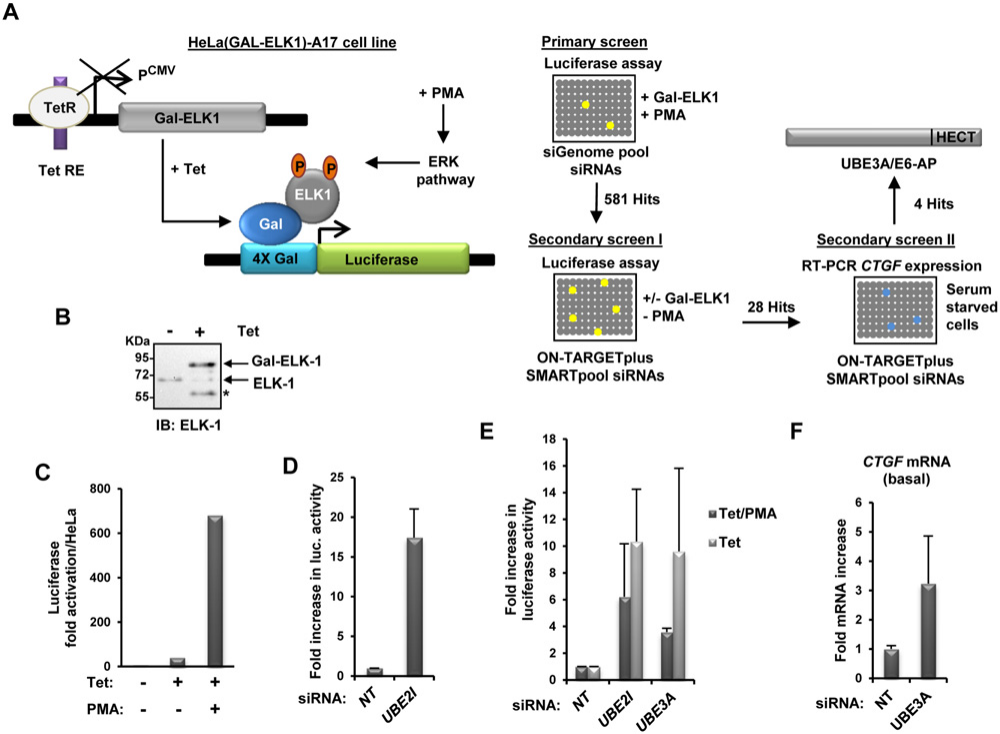
Design and validation of an siRNA screen to identify novel negative regulators of ELK1. **(A)** Left: schematic representation of the two transgenes inserted in the HeLa Flp-In double stable cell line [HeLa(GalELK1-Luc-A17)] created for the siRNA screen. GAL-ELK1 expression is induced by adding tetracycline (Tet) to cause release of the Tet Repressor protein (TetR). Right: diagram of the workflow of the genome-wide siRNA screen and secondary screens I and II. **(B)** Western analysis of the inducible expression of Gal-ELK1 in the stable cell line HeLa(GalELK1-Luc-A17). Where indicated, Gal-ELK1 was induced by the addition of Tet and ELK1 was detected by immunoblotting (IB) using an ELK1 antibody. The positions of bands representing endogenous ELK1 and the Gal-ELK1 fusion protein are indicated. The asterisk likely represents a GAL-ELK1 degradation product. **(C-E)** Luciferase reporter gene assays in the HeLa(GalELK1-Luc-A17). (**C**) Luciferase activity was analysed after treatment of the cells with Tet or Tet and PMA for 5 hours. Data are shown as the fold increase in luciferase activity compared to the parental HeLa Flp-In cell line. **(D)** Luciferase activity following transfection with a non-targeting siRNA (siNT) or a siRNA directed against UBE2I. Data are shown as the average of three independent experiments and represent the fold increase in luciferase activity relative to siNT control (taken as one). Error bars are standard deviation (SD). **(E)** Luciferase activity in cells transfected with the indicated siRNAs and treated with PMA and/or Tet where indicated. Luciferase readings are shown relative to siNT control for each treatment (taken as one)(n = 2). Error bars show standard error (SE). **(F)** RT-qPCR analysis of *CTGF* mRNA expression in serum starved HeLa(GalELK1-Luc-A17) cells transfected with the indicated siRNAs (n = 2). Error bars show SE.

HeLa-Flp-In cells were grown in DMEM supplemented with 10% foetal bovine serum. HeLa-Flp-In cells were used to create double stable cell line expressing Gal-ELK1 full length fusion protein under a tetracycline inducible promoter and a luciferase reporter gene downstream of five Gal4 binding sites. First the stable cell line inducibly-expressing Gal-ELK1 was created by co-transfecting pAS2071 with the Flp recombinase encoding plasmid pOG44 (Invitrogen) into HeLa cells containing a FRT recombination site and the T-REx tetracycline controlled repressor (HeLa-Flp-In; kindly provided by Stephen Taylor). Hygromycin resistant cells were selected, expanded for 24 hours after transfection and maintained as polyclonal lines. Expression of the transgene was induced by treating the cells with 1 μg/ml tetracycline (Sigma) for 24 hours. Then to generate the luciferase reporter cell line, HeLa(GalELK1) cells were transfected with the linearised plasmid pAS4021. Puromycin resistant cells were selected, expanded as single clones and tested for luciferase expression and response to PMA treatment. The A17 clone, HeLa(GalELK1-Luc-A17) was retained as this showed low basal level luciferase activity and high levels of inducibility with phorbol 12-myristate 13-acetate (PMA) (see Fig 1C).

C33A were grown in RMPI supplemented with 10% FBS. SKGI were grown in DMEM containing 10% FBS and 1x Glutamax. MEFs (wild-type, Ube3a-/- [11] and 16E6/E7 stable lines [12]) and SW756 were grown in high glucose DMEM supplemented with 10% foetal bovine serum. MCF10A (obtained from ATCC; CRL-10317) were maintained in DMEM/F12 containing 5% horse serum, 20 ng/ml EGF, 10 μg/ml insulin, 100 ng/ml cholera toxin and 0.5 μg/ml hydrocortisone. SY5Y cells were grown in DMEM/F12 supplemented with 10% foetal bovine serum. Plasmids were transfected using X-treme DNA (Roche) following the manufacturer’s instructions. siRNAs were transfected using Dharmafect1 (Dharmacon), or Lipofectamine 2000 (Life Technologies) for MCF10A cells, following the manufacturer’s instructions. Where indicated, cells were grown in low (0.5%) serum for 48 hrs and were treated with 10 nM PMA, 1.8 ng/ml epidermal growth factor (EGF), 10 μM forskolin/ 0.5 mM 3-isobutyl-1-methylxanthine (IBMX), 10 μM MEK inhibitor U0126 or the control solvent dimethyl sulphoxide (DMSO).

### RNAi and luciferase reporter gene assays

siGENOME pools, ON-TARGETplus SMARTpools, individual ON-TARGETplus and customer designed (for E6) siRNAs duplexes were purchased from Dharmacon.

For luciferase assay HeLa(GalELK1-A17) cells were grown in phenol red-free DMEM. Cells were lysed by adding home-made 2X Lysis-luciferase substrate buffer 48 hours after transfection. Luminescence was measured using an Orion microplate Luminometer (Berthold) plate reader.

### siRNA library screen

For the primary screen, HeLa(GalELK1-A17) cells were reversed transfected using Dharmafect1 and the Human siGenome library (Dharmacon). Cells (11,000 cells/well) were grown in white 96-well plates containing 2 μM siRNA, in phenol-red free DMEM supplemented with 5% foetal bovine serum. The day after transfection cells were serum starved and expression of Gal-ELK1 was induced by addition of 1 μg/ml tetracycline to the media. Luciferase activity was measured 48 hours post-transfection after treatment with 10 nM PMA for five hours. A Biomek robotic system (Beckman Coulter) and Wellmate (Thermo) were used for liquid handling and cell seeding, respectively. Each plate contained the following controls in duplicate, siUBE2I, siGAPDH, siCHD3, siPARP1, siLuciferase and non-targeting siRNA. The quality of each plate was assessed by the luciferase activity measured in the control samples. Luciferase data was normalised and median absolute deviation (MAD) of each plate was calculated. Positive hits were those whom had at least five MAD above the mean. ON-TARGET plus SMARTpools were used for the secondary screens. The first secondary screen measured luciferase activity and was performed as described for the primary screen but cells were either treated or not with PMA. The second secondary screen was carried out in the parental HeLa Flp-In cell line under serum starved conditions. Expression of the ELK1-regulated gene *CTGF* was analysed by RT-qPCR 48 hours after siRNA transfection. Positive hits were those that upon knock down increased *CTGF* expression of at least 2 standard deviations (SD) above the non-targeting control siRNA.

### Western blotting and microscopy

Western blots were carried out using the primary antibodies; P-ERK (Cell Signaling), ERK (Cell Signaling), Tubulin (Sigma), UBE3A (Bethyl Laboratories), ELK1 (Epitomics) and p53 (Calbiochem). Proteins were detected using infrared dye-conjugated secondary antibodies (LI-COR Bioscience, IRDye 800CW and IRDye 680LT). The signal was collected with a LI-COR Odyssey Infrared Imager. Data were quantified using Odyssey software (LI-COR Bioscience, Odyssey Infrared Imaging system application software version 3.0.25). For microscopy, cells were grown on coverslips and fixed in 3.7% Paraformaldehyde for 15 minutes. DNA was staining with Hoechst. Images were acquired on a Delta Vision RT (Applied Precision) restoration microscope using a 100x/1.40 Plan Apo objective. The images were collected using a Coolsnap HQ (Photometrics) camera with a Z optical spacing of 0.2 μm. Raw images were then deconvolved using the Softworx software and maximum intensity projections of these deconvolved images are shown in the results.

### RNA isolation and RT-qPCR

For the secondary screen II total RNA was extracted using the FastLane Cell Multiplex kit (Qiagen) following the manufacturer’s instructions. For the rest of the experiments total RNA was isolated using the RNAeasy plus Kit (Qiagen) following the manufacturer’s instructions. RT-qPCR was performed using 30 ng of total RNA and the QuantiTect SYBR GREEN RT-PCR kit (Qiagen) according to the supplier’s protocol. Data were analysed by Qiagen Rotor-Gene Q Series software and presented after normalisation against the control gene *GAPDH*. The following primer pairs were used for RT-PCR experiments: CTGF, ADS2335/ADS2336 (5’- GAAGAGAACATTAAGAAGGGCA/GCGATTCAAAGATGTCATTGTC-3’), EGR2, ADS2339/ADS2340 (5’- GAGATACCATCCCAGGCTC/ TGATCATGCCATCTCCGG3’), FOS, ADS1690/ADS1691 (5’- AGAATCCGAAGGGAAAGGAA/CTTCTCCTTCAGCAGGTTGG-3’), GAPDH, ADS2184/ADS2185 (5’- ACAGTCAGCCGCATCTTCTT/ TTGATTTTGGAGGGATCTCG -3’), PTGS2, ADS3383/ADS3384 (5’-TGTGTTGACATCCAGATCAC/ GGAAGGGCTCTAGTATAATAGG-3’), UBE3A, ADS3405/ADS3406 (5’- CTGTTCTGATTAGGGAGTTCTGG/ ATGTAGGTAACCTTTCTGTGTCTG -3’) and 18E6 ADS3597/ADS3598 (CACGGAACTGAACACTTCAC/ ATGCGGTATACTGTCTCTATACAC).

## Results

### An RNAi screen to identify negative regulators of ELK1 activity

It has previously been established that SUMO conjugation negatively affects the transactivation activity of ELK1 [13]. To identify novel negatively acting ELK1 regulators, we devised a siRNA-based screen. First, we established a reporter HeLa cell line which contained a fusion protein containing the Gal4 DNA binding domain coupled to full-length ELK1 under the control of tetracycline-inducible promoter to create HeLa(GalELK1) cell lines (Fig 1A, left). This allows the production of Gal-ELK1 fusion protein upon addition of tetracycline to the culture media (Fig 1B). Next we integrated a luciferase reporter gene, driven by four Gal4 binding sites into this cell line. We tested several lines, and continued with the HeLa(GalELK1-Luc-A17) line. In HeLa(GalELK1-Luc-A17) cells little luciferase activity is generated under basal conditions, but a moderate increase is observed upon induction of Gal-ELK1 expression by tetracycline addition, followed by a substantive increase in reporter gene activity following PMA treatment (Fig 1C). Moreover, depletion of UBE2I/UBC9 levels led to increased ELK1 activity as expected from its role in depositing SUMO on substrates (Fig 1D).

Next we performed a primary screen using siRNA pools (siGENOME) in a 96 well format directed against 21,122 different gene products, in the HeLa(GalELK1-Luc-A17) cell line under conditions where ELK1 was activated by PMA treatment (Fig 1A, right). This primary screen was designed to identify siRNA pools that encoded negative regulators of ERK-mediated ELK1 activation and yielded 581 hits where ELK1 activity was elevated >5 absolute deviations above the median in one experiment (or in both experiments when performed in duplicate). We then performed a secondary screen using different pools of siRNAs (SMARTpool) against these hits and assayed ELK1 activity in the absence of PMA stimulation to identify siRNA pools that affected basal levels of ELK1 activity. This resulted in the identification of 28 hits which caused elevated luciferase activity of >1.5 fold in both duplicate samples. Finally we tested these 28 hits for their effect on the basal transcription levels of the ELK1 target gene *CTGF*. Four of these resulted in >1.5 fold increases in basal *CTGF* levels. Little is known about the functions of two of these; *LOC343263*/*MYBPHL* and *DKFZP564K1964*/*TMEM98*. Depletion of a third hit, *CASP8AP2*, resulted in greatly elevated reporter activity even in the absence of tetracycline-induced Gal-ELK1 expression (data not shown). We therefore focussed on the fourth hit *UBE3A* which encodes an E3 ubiquitin ligase whose mis-regulation has previously been associated with the developmental disorder Angelman syndrome [9]. We verified the effect of depleting *UBE3A* on the activity of the Gal-ELK1 fusion protein in the presence of SMARTpool siRNA (Fig 1E) or individual deconvoluted siRNA duplexes (Fig S1A and S1B in S1 File). In both cases depletion of UBE3A caused upregulation of the transactivation capacity of the GAL-ELK1 fusion protein. This increased activity was seen under both basal and PMA activated signalling conditions. Similarly, we were able to verify the effect of depleting UBE3A on *CTGF* expression and found that enhanced basal expression of endogenous *CTGF* was observed in the presence of SMARTpool siRNA against *UBE3A* (Fig 1F).

### UBE3A loss affects immediate-early gene activation kinetics

**Fig 2:**
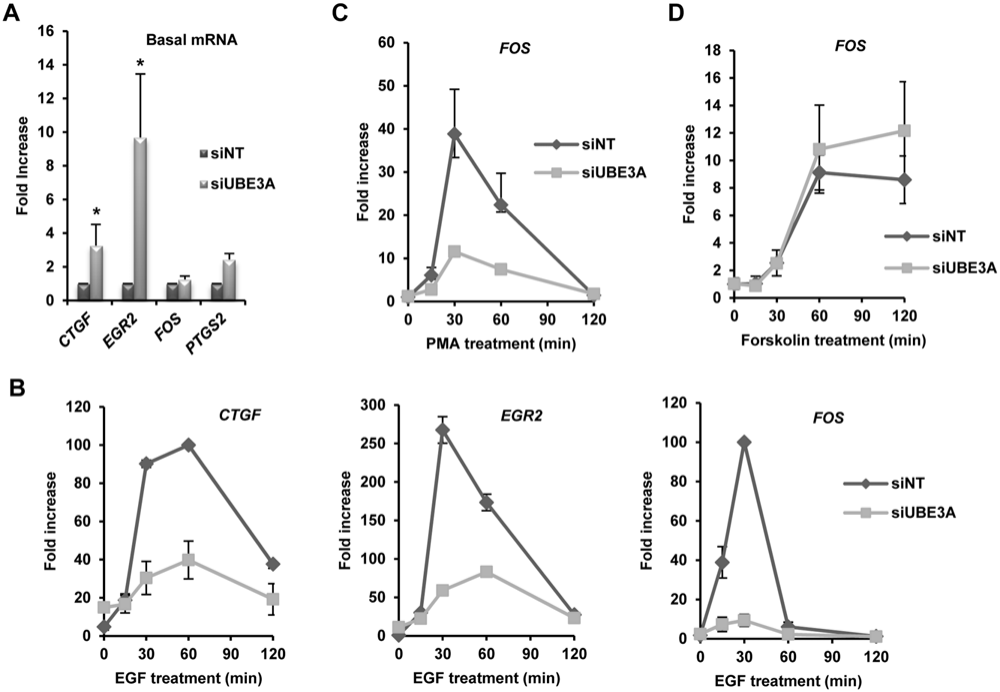
Depletion of UBE3A alters the expression of immediate-early genes. **(A)** RT-qPCR analysis of mRNA expression of the indicated IEGs in serum starved HeLa(GalELK1-Luc-A17) cells treated with control non-targeting (NT) or *UBE3A* siRNAs. Fold increase over the siNT treated control cells (taken as 1) is shown. Data are the average of three independent experiments or two experiments for *PTGS2*. Error bars represent SD or SE for *PTGS2*. Asterisks (*) denote p<0.05 in student’s t-test. **(B)** RT-qPCR analysis of mRNA expression of the indicated IEGs over a time course of EGF stimulation. mRNA levels were measured by RT-qPCR. Values were normalised against GAPDH and fold increases were calculated relative to the zero time point in the siNT treated control (taken as 1). Data are the average of two independent experiments. Error bars represent SE. **(C** and **D)** RT-qPCR analysis of *FOS* expression in siNT control or UBE3A depleted cells after PMA (**C**) or forskolin (**D**) treatment at the indicated time points. Values were normalised against *GAPDH* and fold increases were calculated relative to the zero time point in the siNT treated control (taken as 1). Data are the average of two independent experiments. Error bars represent SE.

Having established that UBE3A dampens down ELK1 transcriptional activation function and the basal level of *CTGF* expression, we next explored whether UBE3A also plays a role in regulating the basal expression levels of other ELK1 target genes like *EGR2*, *FOS* and *PTGS2* that are directly bound and regulated in HeLa cells [14]. Both *EGR2* and *PTGS2* behaved in a similar manner to *CTGF*, and their basal levels were increased upon UBE3A depletion (Fig 2A). This is consistent with a role for UBE3A in maintaining low level ELK1 transcriptional activity. Next we investigated whether depletion of UBE3A would affect the induction kinetics of *FOS*, *EGR2* and *CTGF* following treatment with the ERK pathway activator EGF. Unexpectedly, in all cases, the levels of transcriptional induction were severely attenuated (Fig 2B). To establish whether this effect was specific to EGF stimulation, we also tested *FOS* expression following UBE3A knockdown after stimulation with PMA which also activates the ERK pathway or forskolin which signals via the PKA pathway. Loss of UBE3A caused dampened activation by PMA (Fig 2C) whereas little effect was seen on forskolin-mediated *FOS* activation (Fig 2D). This loss of inducibility likely arises due to ELK1 being activated at basal levels, and therefore not responsive to further activation upon ERK pathway stimulation.

These results therefore indicate that in addition to causing a general increase in basal level expression of immediate-early genes, UBE3A loss specifically affects ERK-pathway mediated signalling. Reciprocally, these results indicate that UBE3A normally acts to suppress the basal level activity of several immediate-early genes but permits robust activation in response to ERK pathway activation.

### UBE3A loss causes enhanced activation of ERK

**Fig 3:**
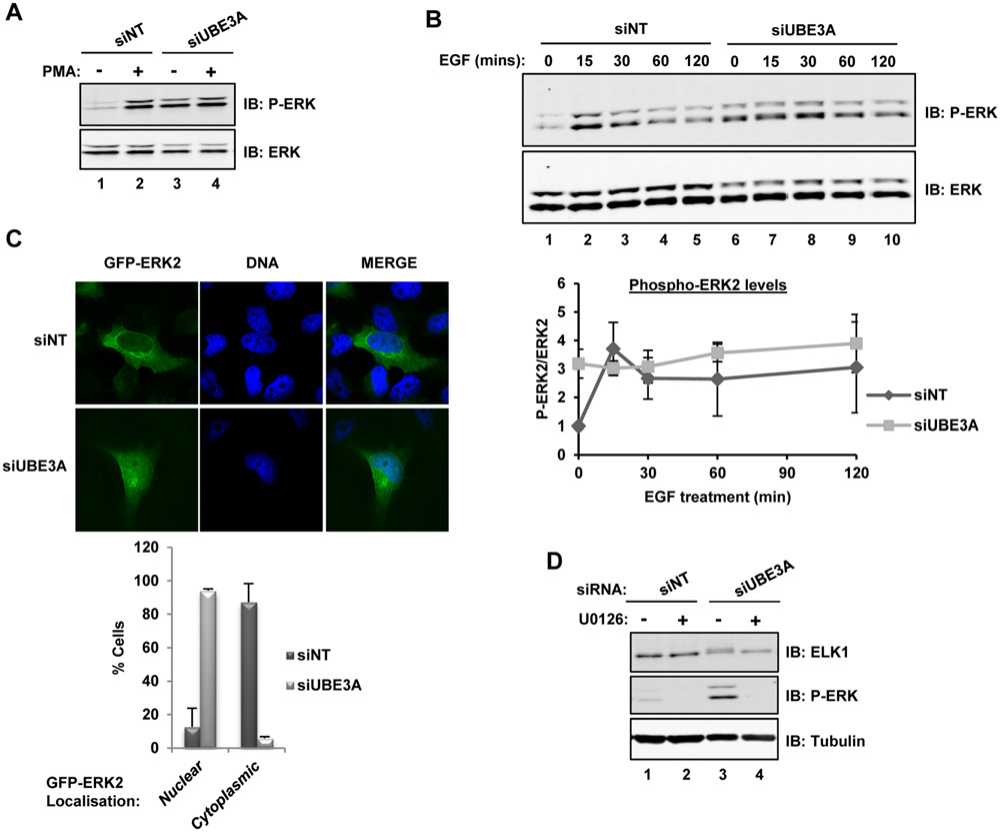
UBE3A depletion alters the kinetics of ERK phosphorylation. **(A** and **B)** ERK phosphorylation status in siNT control or UBE3A depleted cells was analysed by immunoblotting (IB) using an anti-phospho (top; P-ERK) or anti-total (bottom) ERK antibodies. Cells were either serum starved or stimulated with PMA for 15 min (**A**) or EGF for the indicated times (**B**). Graph shows the quantification of the data in (B). Data are shown for ERK2 and are presented relative to phospho-ERK levels in siNT treated control cells at time point zero. **(C)** Top: The cellular localisation of ERK after depletion of UBE3A in serum starved cells was analysed by fluorescent microscopy. ERK2, fused to green fluorescent protein, was transfected in HeLa cells after treatment with siNT control or after depletion of UBE3A. DNA was detected by Hoechst staining. Projections of deconvolved images and a merge of the two signals (DNA, blue and GFP-ERK, green) are shown. Bottom: Quantification of the cellular localisation of ERK in control (siNT; n = 164) and UBE3A depleted (siUBE3A; n = 83) cells. (**D**) Western blotting analysis of ELK1 and anti-phospho ERK (P-ERK) in cells treated with NT control or UBE3A siRNA pools. The MEK inhibitor U0126 was added for 30 mins where indicated. Tubulin IB was used as loading control.

One potential mechanism to explain the increases in basal expression of many IEGs seen upon UBE3A depletion, would be a function of UBE3A in acting upstream from ELK1 on the activity of the ERK pathway. We therefore tested whether depletion of UBE3A affected ERK activation levels in serum starved HeLa-Flp-In cells. As expected, PMA stimulation caused an increase in the levels of the active phosphorylated form of ERK from a low basal state in cells treated with a non-targeting siRNA (Fig 3A; lanes 1 and 2). In contrast, upon UBE3A depletion, the basal levels of ERK activation were already elevated to maximal levels and no further increases were observed following PMA treatment (Fig 3A, lanes 3 and 4). Importantly, increased basal levels of ERK activation were also observed when we deconvoluted the siRNA pools to individual duplexes (Fig S1C and S1D in S1 File). Similar effects on ERK activity were observed following EGF treatment, and UBE3A depletion caused both an elevated basal ERK1/2 activation, and a diminution of the transient increase in ERK phosphorylation (Fig 3B). In addition to the HeLa-Flp-In cells we also tested HeLa and HeLa S3 cells and also observed elevated levels of ERK phosphorylation following UBE3A depletion, albeit to a lower level (Fig S1 in S1 File). Active ERK is known to transit to the nucleus following EGF stimulation [17]. We therefore asked whether UBE3A depletion would also affect the localisation of ERK. In quiescent cells treated with control siRNAs, ERK is found in the cytoplasm as expected (Fig 3C). However, in cells depleted of UBE3A, ERK is almost exclusively nuclear (Fig 3C). Finally we asked whether UBE3A depletion caused phosphorylation of the ERK-targeted transcription factor ELK1 upon translocation to the nucleus. UBE3A depletion caused a characteristic phosphorylation-dependent supershift in ELK1 mobility (Fig 3D, lane 3). Importantly, this was reversed upon simultaneous treatment with the ERK pathway inhibitor U0126 (Fig 3D, lane 4) consistent with an ERK pathway dependent phosphorylation event.

Together, these results demonstrate that the loss of UBE3A results in constitutive activation of ERK in the absence of upstream signals, and the translocation of active ERK to the nuclear compartment where it promotes phosphorylation of ELK1. Phosphorylation of ELK1 can then lead to activation of the basal levels of its target genes such as *CTGF* and *EGR2* (see Fig. 2A).

### UBE3A dampens ERK signalling through the E6-p53 pathway

**Fig 4:**
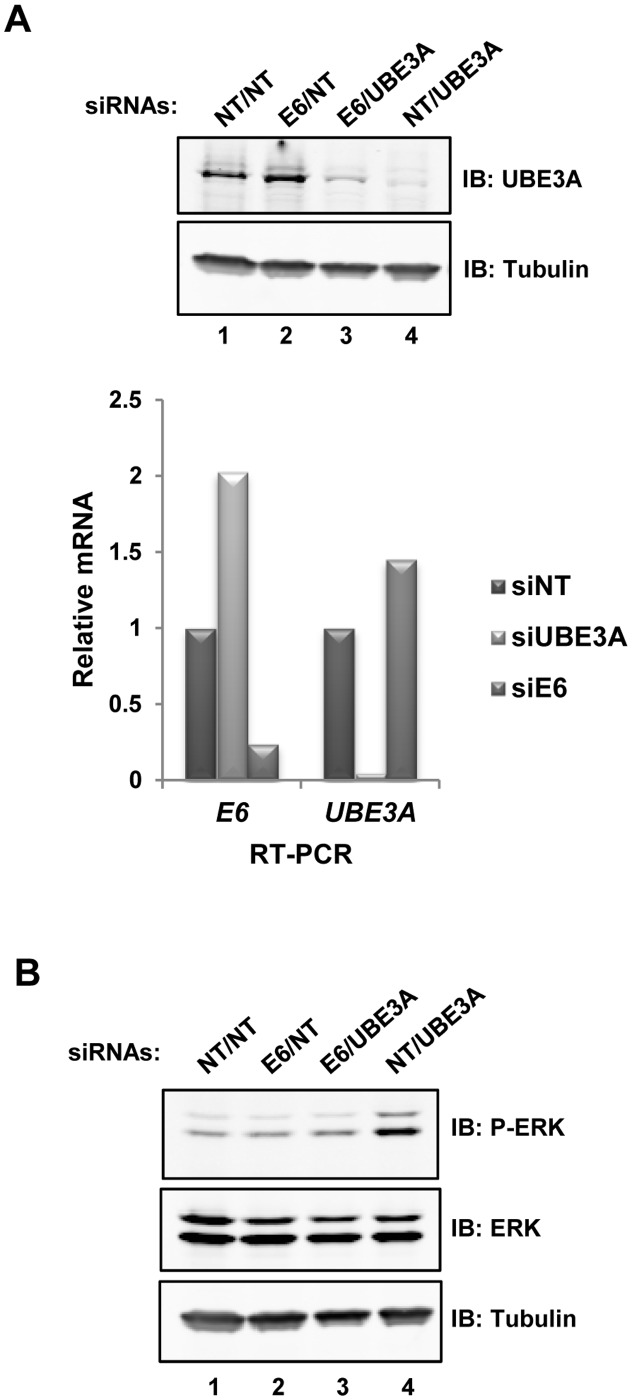
HPV18 E6 is required for enhanced ERK activity in UBE3A depleted cells. HeLa cells were sequentially transfected with different combinations of non-targeting (NT), E6 or UBE3A siRNAs. **(A)** Knock down of UBE3A was verified by immunoblotting (top) and RT-qPCR (bottom). Data is shown relative to siNT control (taken as 1). **(B)** ERK phosphorylation status in siNT control, UBE3A or E6 depleted cells was analysed by immunoblotting (IB) using an anti-phospho (top; P-ERK) or anti-total (bottom) ERK antibodies. Tubulin IB acts as a loading control.

Having established a role for UBE3A in dampening basal ERK signalling in HeLa-derived cells, we examined whether we could observe a similar role in other cell types. However, basal levels of ERK activation remained unaltered following UBE3A depletion in both breast epithelial-derived MCF10A cells and neuroblastoma SH-SY5Y cells (Fig S2A and S2B in S1 File). We also examined mouse knockout MEFs lacking Ube3a, and found no evidence for increased basal levels of ERK activation (Fig. S2C in S1 File), consistent with previous studies [18]. Given these results, we suspected that there were specific molecular changes in HeLa cells which may have rewired UBE3A function. UBE3A was initially identified as the human papillomavirus E6 binding protein E6-AP and the association of UBE3A with E6 has been reported to alter the normal cellular functions of UBE3A [7, 8]. Since HeLa cells express the human papilloma virus-derived oncogenic E6 protein, we asked whether depletion of the UBE3A binding partner E6 would rescue the effect of UBE3A depletion. Efficient depletion of UBE3A and/or E6 mRNA and UBE3A protein was achieved (Fig 4A). Depletion of E6 had little effect on basal ERK levels while UBE3A depletion caused an increase in basal levels of ERK activation as observed previously (Fig 4B, lanes 2 and 4). However depletion E6 prior to UBE3A knockdown, dampened the stimulatory effect of UBE3A depletion on ERK activation (Fig 4B, lane 3). Thus in the absence of E6, UBE3A is no longer required to dampen ERK signalling. This suggested a role for E6 in promoting ERK signalling in HeLa cells which is blocked by the presence of UBE3A.

**Fig 5:**
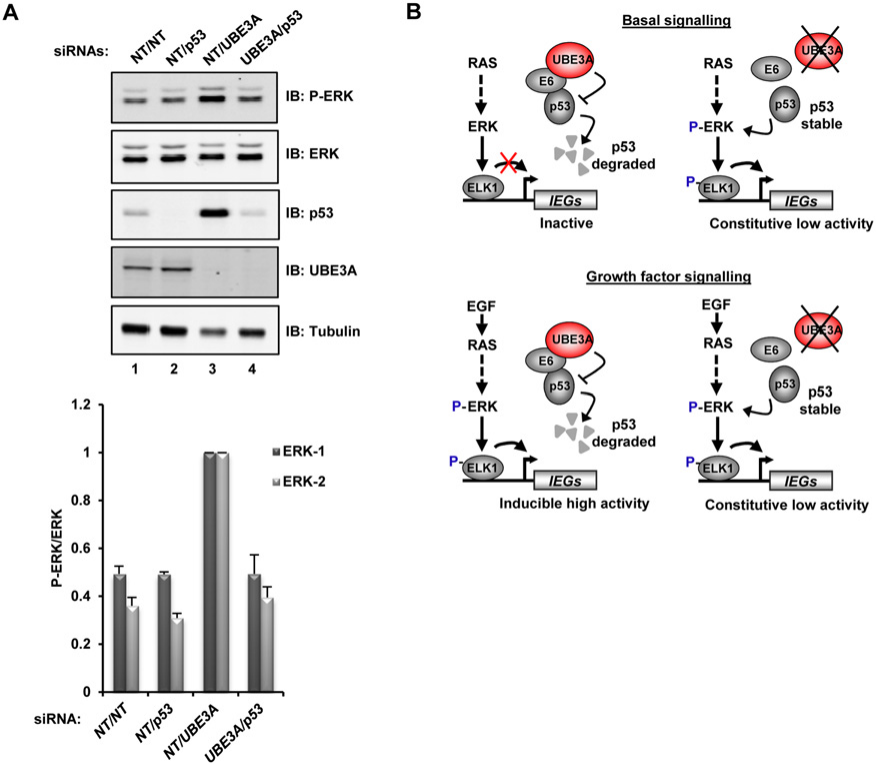
Deletion of p53 prevents activation of ERK in UBE3A depleted cells. **(A)** HeLa cells were transfected with the indicated combinations of non-targeting (NT), p53 or UBE3A siRNAs. Top: immunoblotting (IB) of phosphorylated (P-ERK) and total ERK, p53, and UBE3A. Bottom: quantification of the immunoblot. Data are shown as a ratio of P-ERK to total ERK and is shown relative to NT/UBE3A siRNA transfected cells (taken as 1). Error bars are SE (n = 2). **(B)** Model for how UBE3A acts on the E6-p53 complex to stop p53 causing ERK phosphorylation and activation. In the presence of UBE3A, p53 is degraded and in response to growth factor signalling, ERK is transiently activated, resulting in transient activation of IEGs from a low basal level (left). Removal of UBE3A under basal signalling conditions allows p53 accumulation and liberates p53 to activate basal levels of ERK signalling and low level immediate-early gene (IEG) activation. In the presence of growth factors, no further increases in ERK phosphorylation occur upon loss of UBE3A, and IEG activity remains at a low level (right).

Previous studies have implicated the interaction E6-UBE3A in the degradation of the cellular protein p53. Depletion of E6 or expression of catalytically inactive UBE3A restores p53 expression in the cell [8, 17]. p53 is a tumour suppressor protein and its induction has been shown to activate the MAPK signalling pathway [18]. Therefore, in order to establish whether p53 is also involved in this regulatory loop, we performed combinatorial knockdowns of p53 and UBE3A. Depletion of p53 had little effect on ERK phosphorylation levels while UBE3A depletion caused an increase in both ERK activation and p53 expression levels (Fig 5A, lanes 2 and 3). However, simultaneous depletion of p53 and UBE3A blocked the stimulatory effect of UBE3A depletion on ERK phosphorylation levels (Fig 5A, lane 4). Thus, in addition to the E6 protein, the E6 binding protein p53 is required for the enhanced levels of ERK activation observed following UBE3A depletion. Mechanistically UBE3A therefore acts to dampen down ERK pathway signalling in E6 containing HeLa cells, and E6-p53 interactions appear pivotal in promoting enhanced ERK signalling in the absence of UBE3A which usually acts to shield cells from ERK activation by E6 (Fig 5B).

Finally we asked whether other HPV E6 containing cell lines also exhibited high basal levels of ERK activation upon UBE3A depletion. However, treatment of the HPV18 containing cell lines, SW756 and SKGI with siRNA against UBE3A barely affected ERK phosphorylation (Fig S3A in S1 File). Furthermore, neither human C33A cells nor mouse MEFs ectopically expressing HPV16 E6 protein exhibited large increases in basal ERK phosphorylation levels following UBE3A depletion (Fig S3B in S1 File). Thus although UBE3A works in an E6-dependent manner in HeLa cells, its role in dampening ERK activation levels appears limited to HeLa-derived cell lines.

## Discussion

The mechanisms which are involved in the activation of immediate-early genes in response to growth factor-mediated ERK pathway activation have been extensively studied [4]. However, less attention has been given to how these genes are turned off and how different signalling regimes might affect their activation kinetics. Here we performed a genome-wide siRNA screen in HeLa cells and identified UBE3A as a negative regulator of ERK pathway signalling. Mechanistically, we demonstrated that UBE3A works through its binding partner papilloma virus E6 which targets it to degrade p53. In the absence of UBE3A, p53 is no longer degraded and is able to promote phosphorylation and activation of ERK even in the absence of growth factor stimulation, leading to low level constitutive activation of several immediate-early genes (Fig 5B). In the presence of growth factors, immediate-early genes are robustly activated but once UBE3A is depleted, this activation is severely dampened (Fig 5B). Thus, in the context of E6 transformed HeLa cells, UBE3A plays an important role in maintaining transient ERK signalling responses and the correct expression kinetics of immediate-early genes.

Our study was conducted in human HPV18 transformed cervical cancer HeLa-derived cell lines. We also tested other cell lines but were unable to demonstrate a similar role for UBE3A in dampening basal level ERK signalling (see Figs S2 and S3 in S1 File). This also included other cell lines that contained HPV16 or HPV18 E6 proteins. These seemingly contradictory results could be attributed to the specific mutations observed in cancer cells, where HeLa cells presumably have additional unique mutations not shared by the other cell lines we tested. This is further illustrated by our finding that low basal levels of p53 are observed in HeLa cells and depletion of UBE3A results in increased p53, however, in another HPV18 positive cell line, SKGI, p53 basal levels are not increased upon UBE3A depletion (data not shown). In the context of HeLa cells the presence of the viral E6 transforming protein is essential for the role of UBE3A in dampening basal level ERK signalling. Presumably, without UBE3A, E6 would trigger ERK pathway activation, and UBE3A protects against this. This might be important to viral function, so it can either utilise a normally functioning ERK pathway or avoid the potential detrimental effects of having a constitutively active ERK pathway. Activation of ERK in the absence of UBE3A requires the presence of p53, a protein that is promoted for degradation by E6-UBE3A [8, 17]. Importantly, other studies have demonstrated that p53 can affect ERK pathway signalling [18]. Therefore it is possible that UBE3A might function in other cell types where it is involved in p53 turnover.

Loss of UBE3A function is associated with Angelman syndrome which is a developmental disorder that leads to neurological defects in the brain [9]. Studies in mouse models of this syndrome have revealed few defects in ERK signalling [16, 19]. Instead, reduced signalling via CaMKII has been observed leading to reduced levels of activation of immediate-early genes like *FOS* [20]. However more recently, it was noted that although basal ERK levels are unaffected, ERK activation following KCl treatment is impaired in hippocampal slices derived from Ube3a deficient mice [19]. In another study, the loss of Ube3a was linked to increased p53 levels in mouse-derived purkinje cells but no links to potential changes in ERK phosphorylation levels were made [11]. Thus, although there are links between Ube3a and p53 stabilisation and ERK pathway activation kinetics in the mouse brain, our findings do not appear to be directly relevant to the defects underlying Angelman syndrome and instead are more likely relevant in HPV infected cells.

In addition to insights into viral E6-mediated cellular pathway rewiring, our study contributes to a growing body of knowledge about the impact of constitutive ERK signalling on cellular responses to growth factors. Many cancers exhibit elevated levels of ERK pathway signalling through the constitutive activation of upstream components such as Ras or Braf [21] but the impact of this continuous signalling on downstream gene activation is largely unknown. Interestingly, in rat fibroblasts transformed with adenoviral E1A and oncogenic cHA-Ras, higher basal levels of active ERK are observed which are not further activated by serum stimulation. This impacts on downstream gene expression kinetics where expression of the immediate-early gene *Fos* is not activated by serum addition [22]. This is analogous to what we observe in the absence of UBE3A in E6-transformed HeLa cells, where increased basal level ERK activation precludes further induction in response to growth factor signalling and reduces the activation of immediate-early genes. This phenomenon does not appear to be limited to virally transformed cells, as constitutively increasing ERK signalling by expression of a constitutively active form of BRaf in mouse NIH3T3 cells also inhibits immediate-early gene activation (EA-M and ADS, unpublished data). Mechanistically, basal level activation of IEGs likely arises in the presence of elevated basal ERK phosphorylation levels due to constitutive phosphorylation and activation of ELK1, and this constitutive activation precludes further stimulation in response of ELK1 activity in response to growth factors and mitogens. Together, these observations emphasise the requirement for transient ERK activation to promote the temporal activation of immediate-early genes. Further studies are required to understand the mechanistic basis to this phenomenon.

## Supporting Information

S1 File This includes Figs. S1 to S3. (PDF)
