# Supplementary material for: The Ubiquitin Ligase UBE3A Dampens ERK Pathway Signalling in HPV E6 Transformed HeLa Cells
Source: PLoS One. 2015 Mar 27;10(3):e0119366. doi: 10.1371/journal.pone.0119366 (PMC4376912; doi:10.1371/journal.pone.0119366)
Supplement: S1 File — (PDF) [file pone.0119366.s001.pdf]

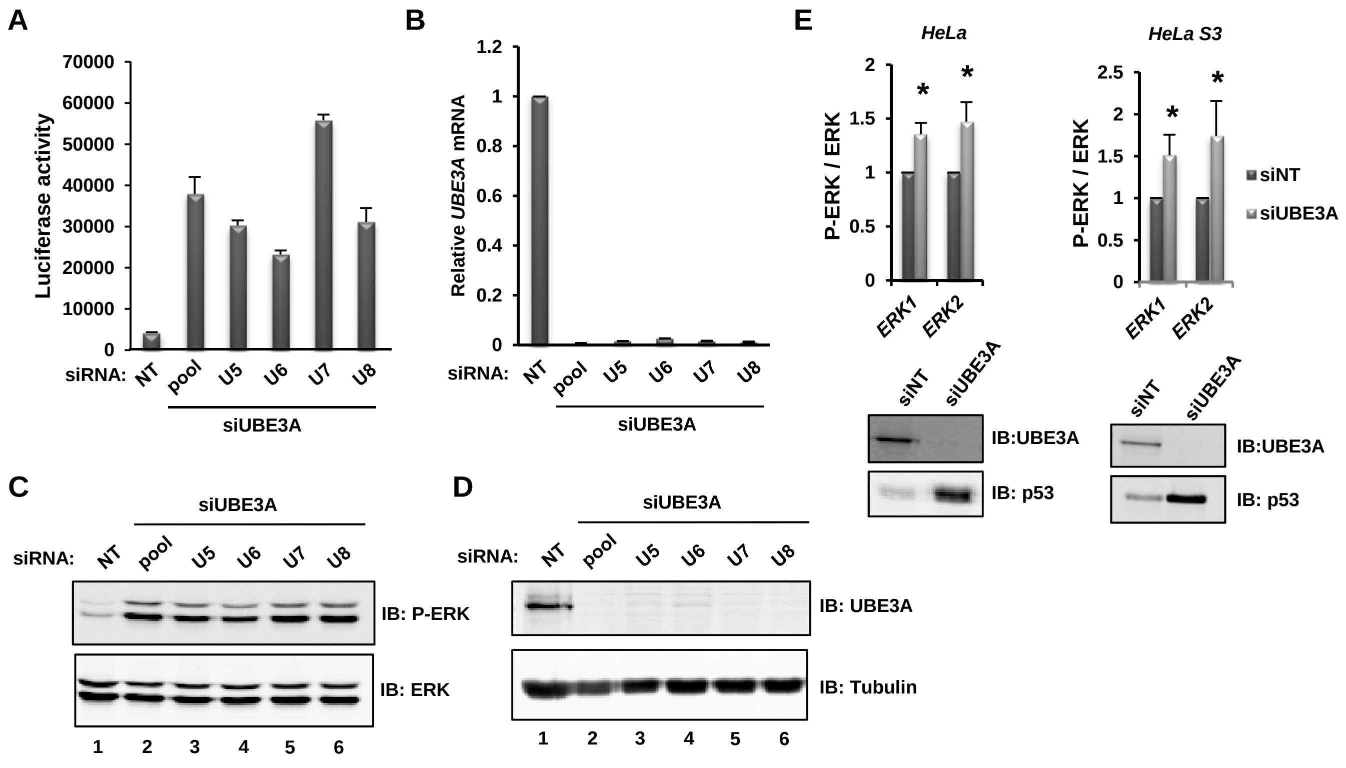

Supplementary Figure S1. Deconvolution of the siRNA pool targeting UBE3A. HeLa(GalELK1-Luc-A17) cells were transfected with non-targeting (NT) siRNA, individual (U5, U6, U7 or U8) or pool (containing U5 to U8) siRNA duplexes against UBE3A. **(A)** Luciferase activity from the Gal-luciferase reporter after depletion of UBE3A using the indicated siRNAs. Relative luciferase activity is shown. Error bars are SE (n=2). **(B)** RT-qPCR analysis of *UBE3A* levels after treatment with the indicated siRNAs. Values are normalised to *GAPDH* and are shown relative to NT siRNA (taken as 1). Error bars are SE (n=2). **(C)** Western blot analysis of ERK phosphorylation status after depletion of *UBE3A* with the indicated siRNAs. Phospho-ERK (P-ERK) and total ERK were detected by immunoblotting (IB). **(D)** Western blot analysis of UBE3A protein levels following treatment of cells with the indicated siRNAs. UBE3A and tubulin loading controls were detected by immunoblotting (IB) with the indicated antibodies. (**E**) UBE3A was depleted by siRNA in HeLa and HeLa S3 cells. Top: the plots show the quantification of independent immunoblots for total and phosphorylated ERK 1 or 2. Data are shown as a ratio of P-ERK to total ERK and are shown relative to non-targeting (NT) siRNA transfected cells (taken as 1). Error bars are SD (n=4 for HeLa and n=3 for HeLa S3). Asterisks (*) denote $p<0.05$ in student’s t-test. Bottom: Depletion of UBE3A was confirmed by immunoblot (IB) of UBE3A and upregulation of p53 with the indicated antibodies.

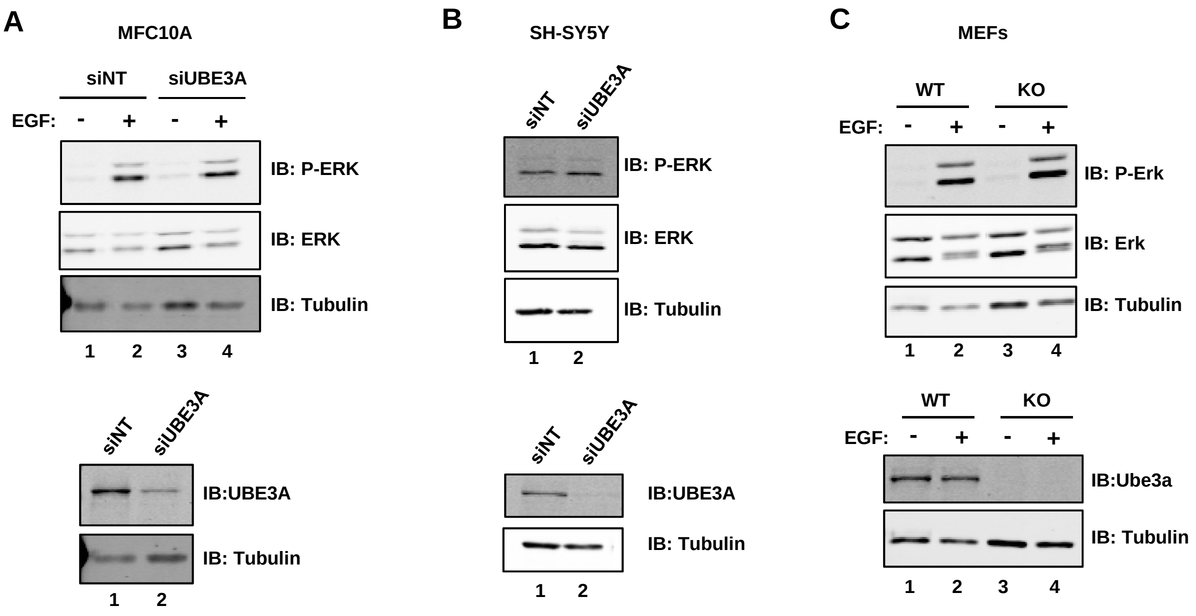

Supplementary Figure 2. **The effect of UBE3A depletion on ERK phosphorylation levels in different cell lines.** (A and B) ERK phosphorylation status after depleting UBE3A in MCF10A (A) or SH-SY5Y (B) cells was analysed by western blotting. After transfection of the non-targeting (NT) or UBE3A siRNAs, cells were grown in low serum and EGF deprived media (MCF10A) or serum free media (SH-SY5Y). Where indicated cells were treated with EGF for 15 minutes. Top: total and phosphorylated ERK (P-ERK) were detected by immunoblotting (IB) with the indicated antibodies. Bottom: UBE3A was detected by immunoblotting (IB). Tubulin was used as loading control. (C) Erk phosphorylation status in wild-type (WT) and *Ube3a* knockout (KO) MEFs was analysed by western blotting. MEFs were grown in serum free media and where indicated cells were treated with EGF for 15 minutes. Top: total and phosphorylated Erk (P-Erk) were detected by immunoblotting (IB) with the indicated antibodies. Bottom: UBE3A was detected by immunoblotting (IB). Tubulin was used as loading control.

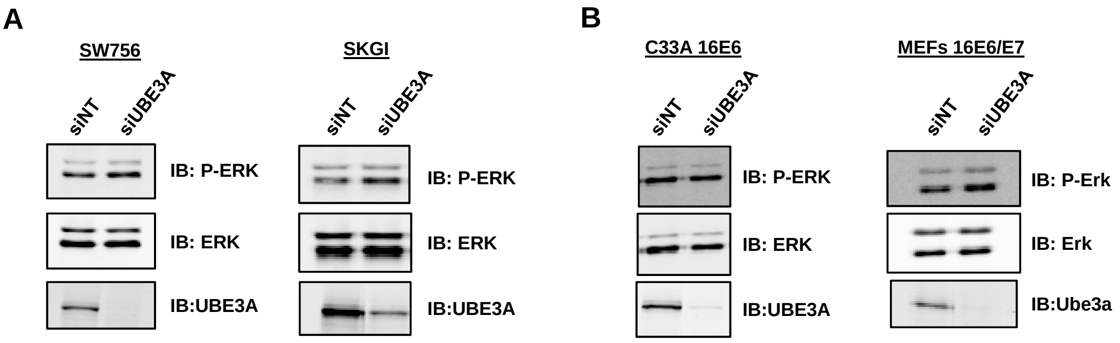

Supplementary Figure 3. The effect of UBE3A depletion on ERK phosphorylation levels in different E6 positive cell lines. (A) ERK phosphorylation status was analysed by immunoblot (IB) in the HPV18 positive cell lines SW756 and SKGI after treating cells with non-targeting (NT) or UBE3A siRNAs. Total and phosphorylated ERK (P-ERK), and UBE3A were detected with the indicated antibodies. (B) Cell lines ectopically expressing HPV16 E6 (C33A E6) or HPV16 E6/E7 (Mefs E6/E7) were transfected with siRNAs to deplete UBE3A. Total and phosphorylated Erk (P-Erk) and Ube3a were detected by immunoblot (IB).
